# SARS-CoV-2 FP1 Destabilizes Lipid Membranes and Facilitates Pore Formation

**DOI:** 10.3390/ijms26020686

**Published:** 2025-01-15

**Authors:** Maria Sumarokova, Rais Pavlov, Tatiana Lavushchenko, Egor Vasilenko, Grigory Kozhemyakin, Oleg Fedorov, Rodion Molotkovsky, Pavel Bashkirov

**Affiliations:** Research Institute for Systems Biology and Medicine (RISBM), Nauchnyi proezd 18, 117246 Moscow, Russiarodion.molotkovskiy@sysbiomed.ru (R.M.)

**Keywords:** lipid bilayer, fusion peptide, SARS-CoV-2, AFM, membrane pore

## Abstract

SARS-CoV-2 viral entry requires membrane fusion, which is facilitated by the fusion peptides within its spike protein. These predominantly hydrophobic peptides insert into target membranes; however, their precise mechanistic role in membrane fusion remains incompletely understood. Here, we investigate how FP1 (SFIEDLLFNKVTLADAGFIK), the N-terminal fusion peptide, modulates membrane stability and barrier function across various model membrane systems. Through a complementary suite of biophysical techniques—including electrophysiology, fluorescence spectroscopy, and atomic force microscopy—we demonstrate that FP1 significantly promotes pore formation and alters the membrane’s mechanical properties. Our findings reveal that FP1 reduces the energy barrier for membrane defect formation and stimulates the appearance of stable conducting pores, with effects modulated by membrane composition and mechanical stress. The observed membrane-destabilizing activity suggests that, beyond its anchoring function, FP1 may facilitate viral fusion by locally disrupting membrane integrity. These results provide mechanistic insights into SARS-CoV-2 membrane fusion mechanisms and highlight the complex interplay between fusion peptides and target membranes during viral entry.

## 1. Introduction

Coronaviruses, including severe acute respiratory syndrome coronavirus 2 (SARS-CoV-2), belong to the family of enveloped viruses, characterized by a viral capsid encased in a lipid bilayer acquired during assembly through budding from host cell membranes [1]. The lipid envelope renders SARS-CoV-2 infection contingent upon membrane fusion—a fundamental process in which the viral envelope merges with cellular membranes to create a continuous lipid bilayer [2,3]. This critical mechanism enables viral capsid disassembly and the subsequent release of viral RNA into the host cell’s cytoplasm.

Membrane fusion, the merging of two lipid-bilayer-bounded compartments, is an energetically demanding process requiring the extensive local reorganization of the bilayer structure, proceeding through the formation of distinct metastable, non-lamellar intermediates [4,5,6]. The initial step involves the formation of a stalk—a characteristic hourglass-shaped structure where proximal membrane leaflets merge while distal leaflets remain separate [7,8]. To complete membrane fusion and establish a continuous aqueous connection between the two compartments, the stalk must transform into a fusion pore [6,9,10,11]. Both these transitions require overcoming substantial energy barriers.

In SARS-CoV-2, this energetically intensive process is mediated by the spike (S) protein, a trimeric class I fusion protein composed of two functional subunits: the receptor-binding S1 and the fusion-mediating S2. The S1 subunit contains the receptor-binding domain (RBD), which specifically recognizes and binds to angiotensin-converting enzyme 2 (ACE2) on the host cells. Meanwhile, the S2 subunit houses the fusion machinery, including the fusion peptide (FP) that is rich in hydrophobic amino acids, heptad repeat regions (HR1 and HR2), and the transmembrane domain [12,13].

The fusion competence of the S protein requires proteolytic processing at two distinct sites: the S1/S2 boundary and the S2′ site. While S1/S2 cleavage occurs during protein biosynthesis, the critical S2′ site, located immediately upstream of the FP, must be cleaved during the entry process [14]. The S2′ proteolysis occurs at Ser816 in SARS-CoV-2, forming a mature N-terminus of the fusion peptide [15]. The cleavage can occur at the plasma membrane via the transmembrane serine protease TMPRSS2 or within the endosomes by cathepsin L, corresponding to the two distinct entry pathways available to SARS-CoV-2 [12,16,17].

Upon receptor engagement and subsequent S2′ proteolytic activation, S1 dissociation triggers extensive conformational changes in S2, resulting in a dramatic transformation from the prefusion spike to the rod-shaped post-fusion S2 subunit. This rearrangement is thought to transition through an intermediate state that exposes the hydrophobic FP, which can readily insert into the hydrophobic core of the target membrane when in proximity [18,19,20].

The final refolding of S2 into its post-fusion state involves the trimerization of HR1 domains and their subsequent assembly with HR2 domains into a six-helix bundle, with the FP serving as an anchor in the target membrane [15,18,21]. This structural transformation generates considerable force, pulling the membrane-anchored FPs toward the viral transmembrane domain and bringing the opposing membranes into close proximity [12,22]. This mechanical action overcomes hydration repulsion, thereby initiating the fusion cascade [23].

The fusion peptide consists of a 41-amino-acid sequence that begins at Ser816 [24,25] and comprises approximately equal-length sections FP1 and FP2, located at the N- and C-terminus, respectively. This sequence features motifs that are highly conserved among coronaviruses [26,27], making it a potential antigen for monoclonal antibodies targeting the fusion peptides across all human coronaviruses [28]. Such antibodies have the capacity to neutralize viruses by inhibiting their fusion with host cell membranes, highlighting the fusion peptide as a promising candidate epitope for next-generation coronavirus vaccine development.

Despite its therapeutic potential, the precise role of the fusion peptide in the membrane fusion process remains incompletely understood. While this peptide likely acts as a molecular ‘hook’ to anchor the viral machinery to the target membrane, it also functions as a membrane-modulating agent [24,29,30,31,32]. Evidence indicates that the isolated SARS-CoV-2 fusion peptide deeply penetrates the lipid bilayers upon membrane interaction [25,30,33]. Nuclear magnetic resonance (NMR) studies have revealed that FP, which exists in a dynamically disordered state in aqueous environments, undergoes significant structural changes upon membrane insertion. Membrane interaction induces a conformational rearrangement characterized by two amphipathic helices in the N-terminal half (FP1) and a single polar helix in the C-terminal half (FP2) [25,33]. FP1 resides within the hydrophobic core of the lipid bilayer, while FP2 interacts with the polar lipid heads [24,25]. Furthermore, isolated FP1 and FP2 demonstrate similar behavior when studied independently [30].

The precise influence of the SARS-CoV-2 fusion peptide on lipid order is complex and context-dependent, with conflicting results reported in the literature. Some studies have observed a decrease in lipid order and an increase in membrane fluidity upon FP interaction [29,30], while others have reported an increase in lipid order in specific membrane regions [24]. These discrepancies underscore the sensitivity of FP-induced membrane changes to experimental conditions, including pH, calcium concentration, and specific lipid composition [30,34]. Nevertheless, it has been consistently observed that FP1 insertion significantly alters the lipid packing in the vicinity of the insertion site. Recent studies indicate that this insertion involves the incorporation of polar and charged amino acids into the hydrophobic core, resulting in localized membrane narrowing [33]. This wedging effect, combined with the introduction of charged and polar moieties, can potentially induce spontaneous membrane poration, thereby compromising membrane barrier function.

In the canonical model of membrane fusion, the integrity of merging compartments is preserved, maintaining their volume identity relative to the surrounding environment [23]. However, emerging studies have suggested a fusion pathway in which holes may form in one of the fusing membranes, evolving into a fused pore, including those promoted by viral fusion proteins [35,36]. Experimental evidence indicates that a pore can sometimes develop during the early stages of viral fusion in one of the membranes [37]. Molecular dynamics’ simulations further corroborate that fusion can occur alongside leakage in these initial stages [38]. The formation of pores can inhibit fusion as well [39]; whether a pore aids or hinders membrane merging depends on the depth and geometry of FP insertion [39,40]. Thus, pore formation can facilitate fusion by serving as an alternative pathway for hemifusion as well as the transition from stalk formation to fusion pore development, although it may also inhibit fusion if the metastable pore possesses lower energy than the fusion intermediates.

To further elucidate these mechanisms, we investigated how the FP1 peptide influences membrane stability and organization across three distinct lipid compositions: pure dioleoyl phosphatidylcholine (DOPC) bilayers, DOPC:cholesterol (Chol) mixtures, and a cellular-membrane-mimicking composition (CMM). The CMM contained DOPC, Chol, dioleoyl phosphatidylserine (DOPS), and dioleoyl phosphatidylethanolamine (DOPE) in molar ratios of 35.7:35:8.3:20.9, respectively [41]. Using AFM force spectroscopy, we monitored the membrane rupture dynamics [42,43], while electrophysiology measurements and calcein leakage assays revealed the FP1-induced changes in membrane permeability [44,45]. Our findings demonstrate that FP1 significantly reduces the activation barrier for conductive defect formation in lipid bilayers, with defect size and dynamics strongly dependent on membrane composition. These results suggest a possible role for controlled membrane destabilization in viral fusion, where precisely regulated pore defect formation may facilitate the fusion process.

## 2. Results

### 2.1. Circular Dichroism Analysis of FP1 Peptide: Structural Changes and Membrane Interaction

We synthesized the FP1 peptide (SFIEDLLFNKVTLADAGFIK), corresponding to positions 816–835 of the SARS-CoV-2 spike protein (S). To verify the peptide’s ability to bind to lipid membranes and to assess the changes in its secondary structure during this process, we measured the peptide’s circular dichroism (CD) spectrum in the UV range (190–250 nm). The resulting data were analyzed using the BeStSel web server to predict secondary structure composition and folding patterns [46].

**Figure 1:**
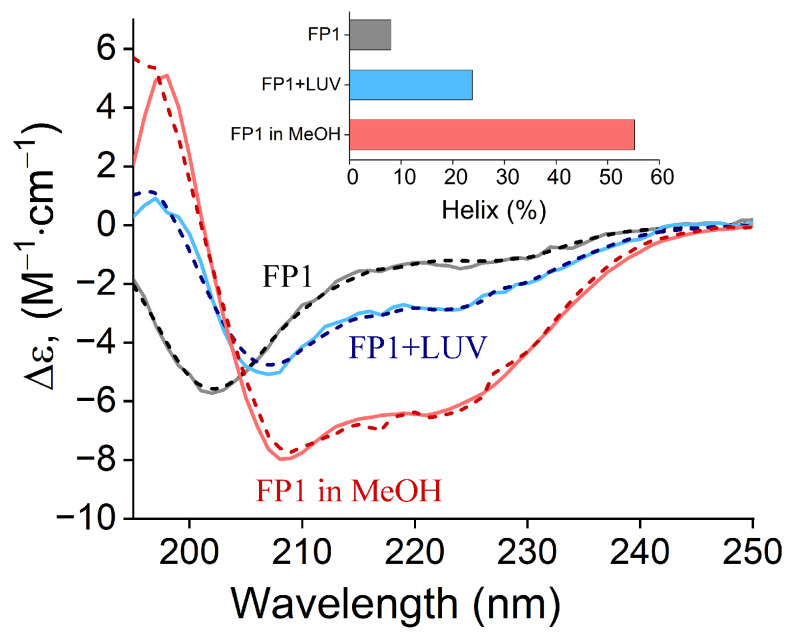
Circular dichroism (CD) spectra of FP1 peptide (25 µM) under different conditions: in 10 mM PBS buffer, pH 7.4 (grey curve); in the presence of DOPC liposomes at peptide:lipid molar ratio 1:1000 (blue curve); in methanol (red curve). Dashed lines represent BeStSel fitting curves used to determine the α-helical content in each condition [46]. The resulting α-helical content values are shown in the inset.

Figure 1 shows the CD spectra of FP1 measured under different environmental conditions, along with their corresponding BeStSel algorithm fits used to calculate peptide structure. In low-calcium and low-ionic-strength conditions (10 mM PBS buffer, pH 7.0), FP1 exhibited a CD spectrum with a negative extremum at approximately 200 nm, characteristic of an unstructured state [47]. BeStSel analysis revealed minimal structuring, with an only 8% alpha-helical content (Figure 1, inset).

A markedly different dichroic signature was observed when FP1 was dissolved in methanol at the same bulk concentration (25 µM). The emergence of two negative extrema at 208 nm and 222 nm indicated substantial helical secondary structure formation [48]. BeStSel analysis confirmed significantly increased structuring in methanol compared to water, with an alpha-helical content exceeding 55%. This methanol spectrum served as a reference, representing FP1’s intrinsic structure in a homogeneous, less polar environment mimicking the lipid bilayer’s hydrophobic core [49].

The CD spectra obtained after incubating FP1 with large DOPC unilamellar vesicles (LUVs, 200 nm diameter, 1:1000 peptide:lipid molar ratio, 1 h) showed a lower α-helicity (27%, BeStSel analysis), reflecting a heterogeneous mixture of aqueous and membrane-inserted conformations. The lack of significant CD changes with added DOPS or cholesterol suggests that FP1’s membrane-bound conformation is primarily determined by its interaction with the hydrophobic core rather than by specific lipid binding. This structural reorganization upon membrane insertion typically affects local membrane properties and may alter membrane permeability, which we investigate in the subsequent sections.

### 2.2. FP1-Induced Membrane Permeabilization: Effects of Lipid Composition

**Figure 2:**
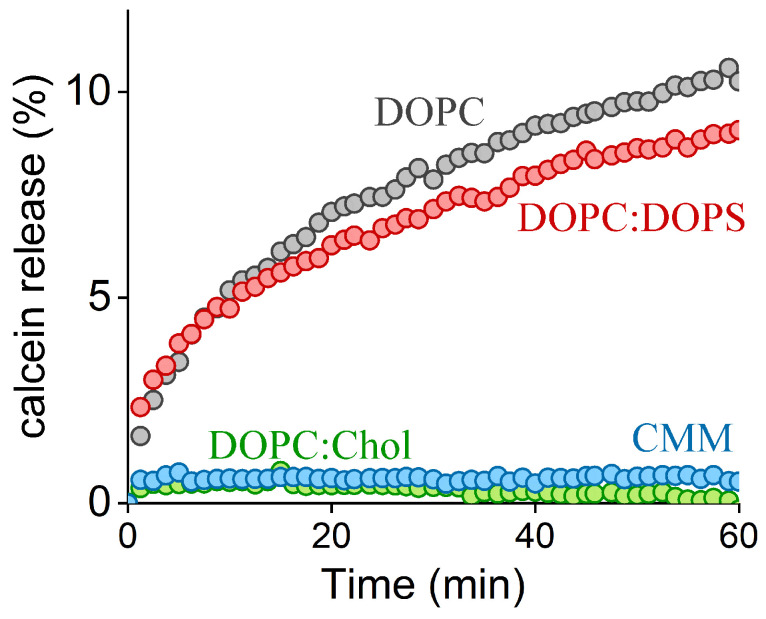
Calcein leakage from large unilamellar vesicles (LUVs) of varying compositions, as indicated in the figure, induced by the addition of FP1 (25 µM) at time t = 0.

To assess the impact of FP1 incorporation on lipid bilayer permeability, we employed a standard calcein leakage assay (Figure 2). LUVs with diameters of 200 nm and varying lipid compositions were prepared and loaded with 20 mM calcein, resulting in near-complete self-quenching. Membrane permeabilization led to calcein release into the external solution, increasing total fluorescence due to the dilution and subsequent dequenching of the dye. We observed slow leakage kinetics in the liposomes composed of DOPC. The addition of negatively charged DOPS did not significantly affect calcein leakage, implying that hydrophobic interactions predominantly govern FP1’s affinity for the membranes. In contrast, no calcein release was detected in the LUVs containing cholesterol, suggesting that cholesterol reduces the membrane disruption caused by FP1.

The observed membrane rupture likely resulted from structural distortions in the lipid bilayer associated with FP1 incorporation, indicating that FP1 enhances the likelihood of pore formation in the vesicle membrane—an effect mitigated by cholesterol. Given that cholesterol has minimal effects on membrane rigidity in DOPC bilayers [50], its protective role could arise through either indirect membrane stabilization or direct peptide interaction. The indirect mechanisms may include (i) cholesterol’s negative spontaneous curvature counteracting local membrane deformations and (ii) rapid cholesterol flip-flop between membrane leaflets compensating for the area imbalance between external and internal lipid leaflets created by FP1. Alternatively, cholesterol might directly modulate FP1 activity through changes in the membrane’s lateral pressure profile, affecting peptide penetration depth and orientation. To distinguish between these possibilities, we employed atomic force microscopy (AFM) to measure the force required to rupture supported lipid bilayers in the presence and absence of FP1.

### 2.3. Quantitative Analysis of FP1-Induced Membrane Destabilization via AFM Force Spectroscopy

**Figure 3:**
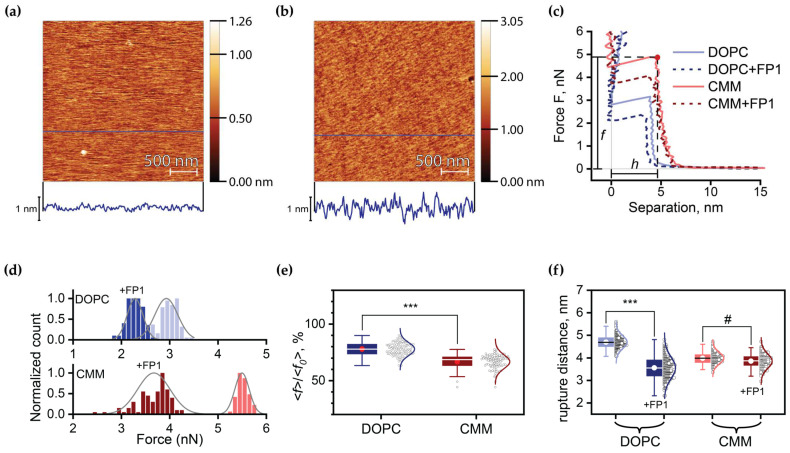
AFM analysis of FP1 effects on breakthrough force of lipid bilayers. (**a**) Height image topography of lipid bilayer without FP1 and (**b**) after FP1 incubation with the corresponding height profiles along the navy lines. Scale bar: 500 nm. (**c**) Representative approach force–distance curves obtained for the studied lipid bilayers before and after FP1 addition. (**d**) Rupture force distribution histograms for lipid bilayers before and after FP1 incubation. (**e**) Relative rupture force of DOPC and CMM lipid bilayers after FP1 exposure, normalized to untreated bilayers. (**f**) Box plot comparison of rupture distances for studied lipid bilayers before and after FP1 incubation. Statistical significance: #—*p* < 0.005; ***—*p* < 0.001.

In our study, we employed atomic force microscopy (AFM) to evaluate the impact of FP1 on the mechanical properties of the supported lipid bilayers on atomically flat mica surfaces (Figure 3a,b). Using force spectroscopy, we recorded force–distance curves during membrane puncture to measure the bilayer’s response to applied stress. To assess the effect of cholesterol, we compared the responses of the DOPC bilayers with those of the CMM, which served as a model for cholesterol-containing membranes.

When the AFM tip applied a point force (*F*) to the membrane surface, the membrane initially exhibited linear elastic behavior, with local deformation proportional to the applied force. However, as *F* increased, the induced stress eventually exceeded the activation barrier for critical defect formation, leading to sudden membrane rupture. This manifested as an abrupt cantilever displacement equal to the membrane thickness, *h*, at which membrane rupture occurred as the tip contacted the underlying substrate.

To ensure consistent loading conditions, all measurements were performed at a fixed cantilever approach speed of 1 μm/s. The resulting force–displacement curves yielded two critical parameters: breakthrough force, *f*, which reflects the energy barrier for critical defect formation, and *h* (Figure 3c). Prior to FP1 addition, the DOPC and CMM bilayers exhibited mean breakthrough forces of <*f*_0_> = 2.9 ± 0.2 nN and 5.5 ± 0.1 nN, with *h* = 4.7 ± 0.3 nm and 4.0 ± 0.3 nm, respectively. All values were in good agreement with the published ones for lipid bilayers, where similar conditions for breakthrough force measurement were employed [43,51].

Incubation with FP1 (2 μM) significantly altered the membrane properties. The root mean square (RMS) surface roughness increased from 0.1 nm to 0.2 nm (Figure 3a,b), indicating successful peptide incorporation into the bilayer. Following FP1 insertion, both compositions showed comparable relative reductions in breakthrough force (Figure 3d,f). Interestingly, while the DOPC bilayer showed a considerable reduction in thickness at which rupture occurred to *h* = 3.6 ± 0.5, the threshold thickness of the CMM bilayers remained unchanged (Figure 3e). The analysis of the force–distance curves for the DOPC bilayers before and after FP1 addition revealed bilayer thinning induced by FP1, as evidenced by the leftward shift in the linear force–displacement region, while FP1 insertion into the CMM appeared to have no considerable impact on its thickness.

Membrane rupture represents a stochastic process occurring through critical defect formation [52]. The probability of defect formation depends on its activation energy ΔU, which is a function of applied force *F*. The force increases linearly with time *t* according to F=Kvt, where *K* is the cantilever spring constant, and *v* is the approach velocity. According to the molecular model developed in [53], the mean breakthrough force <*f*> exhibits a logarithmic dependence on the loading rate:

(1) $$\langle f\rangle ={\alpha f}_{T}ln\left(\frac{Kvln2}{k_{0}\alpha f_{T}}+1\right)$$

where *f_T_* is a “thermal” force, *k*_0_ is the rate of spontaneous formation of a pore in a membrane when *F* = 0, and α is a geometrical factor that is equal 0.5 when the cantilever tip radius (*R*) is much larger than the distance between adjacent lipid molecules. In this model, pressure increases the energy of lipid molecules within a finite activation volume *V* beneath the tip, thereby enhancing the probability of molecular displacement to adjacent pressureless membrane regions, which creates a pore. The thermal force $f_{T}=\frac{4\pi hRk_{B}T}{V}$ represents the force required to compress the activation volume by an amount equivalent to thermal energy *k_B_T*.

**Figure 4:**
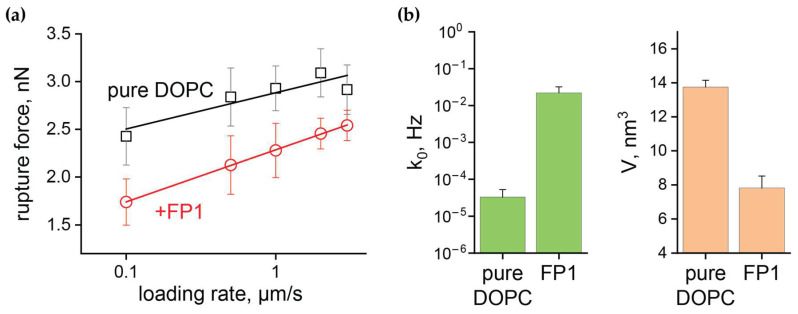
Influence of FP1 on membrane defect formation. (**a**) Dependence of the mean rupture force on the loading rate and (**b**) rate of spontaneous pore formation (*k*_0_) and activation volume (*V*) for pure DOPC bilayers versus DOPC bilayers exposed to FP1.

We investigated the impact of FP1 on the fundamental parameters of membrane stability by measuring breakthrough forces at multiple loading rates. By varying the cantilever approach speed from 0.1 to 3.0 μm/s, we obtained the dependence of breakthrough force on loading rate for the DOPC bilayers before and after FP1 exposure. This allowed us to determine how FP1 affects both *V* and *k*_0_. Figure 4a shows the obtained <*f*(*v*)> dependencies plotted on a logarithmic scale. The linear approximation of these curves, measured using cantilevers with spring constant *K* = 0.12 N/m and tip radius *R* = 10 nm, enabled the determination of the FP1-induced changes in *k*_0_ and *V* (Figure 4b).

Following FP1 incorporation into the lipid bilayer, we observed an ~ 40% decrease in the activation volume *V* and a significant increase in the rate of pore formation *k*_0_, which rose by several orders of magnitude from $k_{0}=3.3\cdot 10^{-5}$ Hz to $k_{0}=2.2\cdot 10^{-2}$ Hz once the fusion peptide was incorporated in the lipid bilayer. Importantly, both the V and *k*_0_ values were in good agreement with the published data [43,54], where measurements were conducted for similar lipid compositions under comparable conditions. Notably, taking into account the reduction in bilayer thickness caused by FP1, the calculated decrease in the area of the activation zone (*S* = *V*/*h*) was less than 25%. This suggests that the dramatic increase in the rate of spontaneous pore formation was the primary factor driving the observed reduction in breakthrough force.

Our AFM studies confirmed that FP1 significantly enhances the rate of spontaneous pore formation, consistent with a substantial reduction in the activation energy barrier for pore nucleation. To further explore the dynamics of FP1-induced membrane destabilization, we employed bilayer lipid membranes (BLMs), enabling the real-time observation of pore formation and evolution. These complementary approaches underscore the central role of FP1 in inducing rapid structural disruption, further elucidating its membrane-destabilizing mechanisms.

### 2.4. Electrophysiological Characterization of FP1-Induced Membrane Defects

The BLM experimented reveal FP1’s ability to induce conducting defects and pores in lipid bilayers under mechanical tension. All measurements were performed under physiological conditions (150 mM NaCl, low Ca^2+^, pH 7.0) with an applied voltage bias of 50 mV. FP1 was added at a concentration of 2 μM to one side of the BLM. We observed several distinct patterns of FP1-induced conducting events (Figure 4a): (i) rupture: instant current increase due to membrane short-circuit; (ii) spike: momentary charge passage through transient defects; (iii) erratic: continuous current through dynamic membrane defects; (iv) multi-level: multiple dynamic conducting defects; (v) step: stable conducting defects or pores; and (vi) channel-like: pores with a defined diameter oscillating between open and closed states.

**Figure 5:**
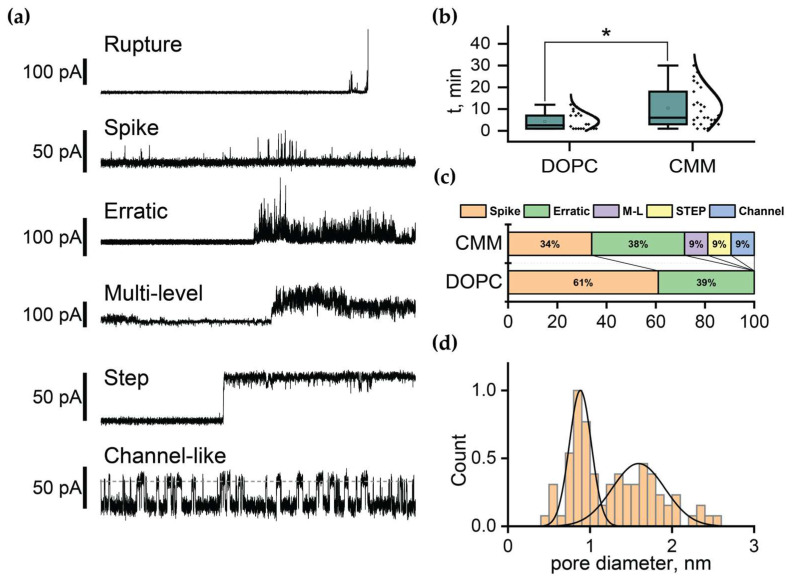
FP1-induced conductance patterns and membrane defects. (**a**) Representative current traces showing characteristic defect types induced by FP1 during planar bilayer lipid membrane (BLM) permeation. (**b**) Average time until the first conductance event caused by FP1 in DOPC and CMM membranes, showing significantly longer latency in CMM membranes (* *p* < 0.05). (**c**) Distribution of membrane defect types present in traces for DOPC and CMM membranes. (**d**) Pore size distribution calculated from step and channel-like current patterns obtained with CMM membranes. Normal distribution peaks correspond to 0.9 and 1.6 nm.

FP1 induced conducting defects more frequently in the DOPC membranes compared to the CMM composition, with CMM membranes exhibiting significantly more complex dynamic behavior (Figure 5b). While FP1 addition to the DOPC membranes primarily led to spikes and erratic conductance increases, culminating in membrane rupture, CMM membranes displayed stable conductance changes characterized by steps and channel-like behavior, suggesting the formation of metastable defects of specific sizes.

The stability difference was quantitatively reflected by the event timing and membrane survival statistics. The CMM bilayers maintained integrity for longer periods during FP1 exposure, with a mean time to first event of 10 ± 8 min compared to 4 ± 3 min for DOPC (*p* < 0.05) (Figure 5c). Similarly, the mean time until complete rupture was extended for CMM (14 ± 12 min vs. 9 ± 10 min), which had a lower probability of membrane rupture during the observation period of 30 min (CMM: 57% vs. DOPC: 74%).

The analysis of the current jumps during pore formation (step and channel-like events, Figure 5a) provided insights into the pore dimensions. Using cylindrical approximation for the forming pores, we found a bimodal size distribution in the CMM membranes, with a prominent peak around 0.9 nm and a second population in the range of 1.4–2.3 nm (Figure 5d). These dimensions align with the theoretical predictions for quasistable pore formation [55]. Notably, only the CMM membranes demonstrated reproducible channel-like behavior with stable pores. In contrast, the DOPC membranes exhibited gradual, long-term conductance increases, indicating the development of large, unstable conducting defects that rapidly progressed to complete rupture.

## 3. Discussion

Our results reveal that SARS-CoV-2 FP1 functions not merely as a membrane anchor but also as an active membrane-modulating agent, with its effects finely regulated by the lipid composition. Upon membrane binding, FP1 undergoes significant structural reorganization, transitioning from an unstructured state in solution to a partially α-helical conformation, as demonstrated by our CD measurements (Figure 1). This structural transition aligns with observations from previous studies using lysolipid micelles [56] and small unilamellar vesicles [24] of varying lipid compositions, suggesting that FP1’s interaction with membranes is primarily driven by hydrophobic rather than electrostatic forces. The high content of non-polar amino acid residues in FP1 facilitates its insertion into the hydrophobic core of the lipid bilayer. Furthermore, its partial α-helical formation in methanol (Figure 1), a relatively non-polar solvent, underscores the importance of hydrophobic environments in driving FP1’s conformational changes. This behavior is consistent with the properties of other amphipathic peptides, which often undergo conformational changes upon membrane interaction [57].

**Figure 6:**
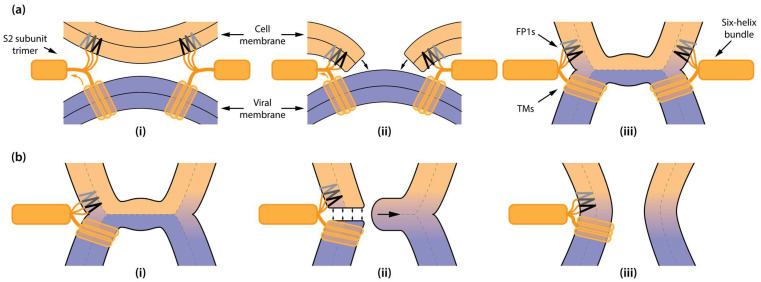
Mechanistic illustration of FP1-mediated membrane perturbation and its possible role in viral fusion conducted by S2 subunit trimers. (**a**) Hemifusion diaphragm formation: (**i**) FP1 insertion into the target membrane (indicated by black and gray arrowheads) and S2 subunit trimer HR1/HR2 domain rearrangement into six-helix bundle bring the viral and cellular membranes into close proximity. (**ii**) FP1-induced local membrane destabilization may facilitate transient membrane breach. (**iii**) Subsequent membrane reconnection forms a hemifusion diaphragm. (**b**) Proposed role of FP1 in fusion pore opening: (**i**) following hemifusion diaphragm formation, FP1—positioned near the S2 subunit transmembrane domain (TM) due to geometric constraints of the post-fusion state—destabilizes the adjacent lipid bilayer, particularly at the trilamellar junction, which is under high elastic stress, making the area susceptible to breach. (**ii**) Localized destabilization triggered by FP1 leads to fusion pore formation. (**iii**) The hemifusion diaphragm subsequently collapses, completing the fusion process.

We further demonstrated that FP1 insertion into unilamellar vesicles leads to membrane-destabilizing effects, compromising the barrier function of the membranes. Calcein leakage assays showed that FP1 induces significant membrane permeabilization. This effect is consistent with earlier reports of FP1 activity [56] and has also been observed for other viral fusion peptides, such as those from influenza and HIV [58,59]. Notably, FP1-driven membrane rupturing has been linked to viral fusion catalysis [30], where it is proposed to represent an alternative molecular mechanism to hemifusion through the formation of a “π-shaped” structure observed for hemagglutinin-induced membrane fusion [36,60]. Unlike the canonical stalk–hemifusion mechanism, the “π-shaped” structure involves transient pore formation within the host membrane as an intermediate step, potentially facilitating membrane merging by increasing the connectivity between proximal monolayers (Figure 6a).

AFM force spectroscopy provided quantitative insights into the mechanical changes induced by FP1 in supported lipid bilayers. Our measurements directly demonstrate that FP1 significantly reduces the point force required to pierce the supported lipid bilayers on mica. According to the molecular model developed in [53], when breakdown conditions (tip radius *R*, cantilever spring constant *K*, and approaching velocity *v*) are maintained constant, a reduction in breakthrough force (Equation (1)) indicates either a decrease in thermal force *f_T_* or an increase in the rate of critical defect spontaneous formation *k*_0_. Our data convincingly demonstrate that the increase in *k*_0_ is responsible for the decrease in membrane piercing strength, implying a substantial reduction in the activation barrier for critical pore formation. This finding is consistent with our observations of increased membrane permeabilization in the calcein leakage experiments.

This mechanism aligns with studies on other amphipathic peptides, such as magainin H2 [61] and the M2AH peptide from influenza A M2 protein [62], which also showed reduced rupture forces upon membrane interaction, indicating weakened mechanical stability. In the case of the HIV-1 fusion inhibitor T-1249 [63], a significant reduction in rupture force was observed, accompanied by increased membrane fluidity and roughness, further supporting a shared mechanism of membrane destabilization among amphipathic peptides, including FP1.

While the rich-in-cholesterol CMM bilayer exhibited higher overall breakthrough forces, consistent with its enhanced mechanical stability, FP1 incorporation led to a significantly much stronger relative reduction in force than for DOPC (Figure 3f). This observation suggests that FP1 lowers the activation barrier for pore formation even in cholesterol-rich membranes, albeit to a lesser absolute value than in pure DOPC bilayers. This interpretation is supported by our planar membrane experiments, where we observed conductive defects in the cholesterol-containing bilayers despite their increased stability. Thus, while cholesterol enhances overall membrane integrity, it does not prevent FP1-induced destabilization but instead delays and modulates the process. The diversity in rupture force distributions observed via AFM further suggests distinct mechanisms of disruption, including the potential formation of metastable pores in cholesterol-containing systems.

Importantly, FP1 may also catalyze fusion through an alternative mechanism involving fusion pore formation in the vicinity of hemifusion structures (Figure 6b). Fusion involves a progression from stalk formation to hemifusion and, ultimately, to the formation of a full fusion pore. Hemifusion structure is a high-energy intermediate displaying significant elastic stress in the lipid bilayer, particularly in the hemifusion diaphragm, trilamellar rim, and its nearby regions. The geometric constraints of the post-fusion state place FP1 in precisely this stressed environment, allowing it to interact with and destabilize the highly strained lipids in this region (Figure 6b). We speculate that cholesterol may play a pivotal role in regulating the threshold tension or elastic stress at which membrane destabilization could occur, facilitating pore formation during late stages when the hemifusion structure is already formed. This regulation helps ensure that under conditions of high elastic stress—when FP1 is likely situated nearby due to geometric constraints—membrane destabilization and pore formation can proceed efficiently.

Our findings suggest that FP1 induces localized structural defects that are particularly effective in areas subject to high elastic stress, such as those surrounding hemifusion diaphragms or stalks, thereby facilitating the emergence of metastable pores that bridge the final stage of viral fusion. This mechanism aligns with our observations of FP1 disrupting membranes under tension, such as in planar bilayers. The high lateral tension in these bilayers mimics the elastic strain present in hemifusion diaphragms during the fusion process. The conductive defects observed in cholesterol-containing planar membranes further suggest that FP1-mediated destabilization persists even in challenging membrane environments, highlighting its functional versatility in catalyzing fusion.

The apparent discrepancy between the cholesterol-mediated protection against calcein leakage in vesicle experiments as well as the observed membrane destabilization in planar bilayers and the reduction in rupture forces by FP1 might be explained by the different membrane conditions. The ability of cholesterol to rapidly flip-flop between monolayers and mitigate the elastic stress associated with FP1 incorporation may be less effective in planar bilayers, as well as in the hemifusion structure, where lateral tension is set by the external reservoir of lipid molecules [64]. In vesicles, where the number of lipid molecules is fixed, the rapid cholesterol redistribution between monolayers prevents significant area asymmetry and the buildup of elastic stress, leading to greater resistance against FP1-induced rupture.

Future studies on FP1 could significantly enhance our understanding of SARS-CoV-2 membrane fusion and pave the way for novel antiviral strategies. Investigating the precise mechanism through which FP1 lowers the activation barrier for pore formation is essential and can be achieved through advanced molecular dynamics’ simulations that incorporate explicit membrane models and detailed FP1–lipid interactions. Furthermore, exploring the interplay between membrane tension and FP1-mediated pore formation is critical; employing techniques such as micropipette aspiration with giant unilamellar vesicles will facilitate the precise control of membrane tension, enabling a quantitative assessment of this relationship and clarifying the conditions under which FP1 exhibits its most effective activity. Collectively, these avenues represent promising directions for future research, ultimately aimed at deepening our understanding of SARS-CoV-2 entry mechanisms and potentially leading to the development of innovative antiviral therapies.

## 4. Materials and Methods

### 4.1. Materials

1,2-Dioleoyl-sn-glycero-3-phosphocholine (DOPC), 1,2-dioleoyl-sn-glycero-3-phosphoethanolamine (DOPE), 1,2-dioleoyl-sn-glycero-3-phospho-L-serine (DOPS), and cholesterol were purchased from Avanti Polar Lipids (Alabaster, AL, USA), stored at −80 °C and used without further purification. Calcein disodium salt was obtained from Serva (Heidelberg, Germany). Phosphate-buffered saline (PBS) tablets from Sigma-Aldrich (St. Louis, MO, USA) were used to produce 1x PBS solutions containing 2.7 mM KCl and 137 mM NaCl at pH = 7.40 in 200 mL of distilled water.

### 4.2. Fusion Peptide (FP1) Synthesis

The SFIEDLLFNKVTLADAGFIK peptide was synthesized via solid-phase peptide synthesis using a PurePep^®^ Chorus automated peptide synthesizer (Protein Technologies, Inc., Stockport, UK). The synthesis employed N-(9-fluorenylmethoxycarbonyl)-Lys (Boc)-Wang resin as the solid phase and Fmoc-protected amino acid derivatives. Amino acid coupling was performed using N,N′-diisopropylcarbodiimide (DIC) with ethyl cyano (hydroxyimino) acetate (Oxyma Pure) as an activator, using a 15-fold excess of Fmoc amino acids relative to carrier capacity.

Fmoc deprotection was achieved using 20% 4-methylpiperidine in N,N-dimethylformamide. After each coupling step, a capping procedure was performed using 5% propionic anhydride in DMF for 10 min. All coupling, capping, and Fmoc-deprotection steps were conducted at 65 °C.

The peptide was cleaved from the resin and simultaneously deprotected using a mixture of trifluoroacetic acid:3,6-dioxa-1,8-octandithiol:triisopropylsilane:anisole:water (183:5:2:5:5 *v*/*v*) for 3 h (5 mL per 100 mg resin). The filtered solution was precipitated with cold diethyl ether, cooled at −20 °C for 30 min, and centrifuged (7000 rpm, 10 min). The precipitate was washed twice with diethyl ether (2 × 25 mL), dissolved in 1 mL of 5% acetonitrile, and lyophilized.

The crude peptide was purified by HPLC using a gradient of 2–100% acetonitrile in water over 25 min (retention time: 12.5 min); the column: was a Reprosil C18, 4.6 × 250 mm, 5 µm, 120 Å. Final yield was 25 mg (31%). MALDI-MS analysis: calculated *m*/*z* = 2240.2094, found [M + H]^+^ = 2241.2153.

### 4.3. Large Unilamellar Vesicle (LUV) Preparation

Large unilamellar vesicles (LUVs) were prepared using a combination of sonication and extrusion methods. Lipids (compositions detailed in Table 1) dissolved in chloroform were mixed at the desired molar ratios. The organic solvent was removed under an argon stream, followed by vacuum-drying for 1 h. The resulting lipid films were hydrated with buffer solutions (specified in the subsequent experimental sections), subjected to 10 freeze–thaw cycles (liquid nitrogen and hot water bath), and then processed further.

For sonication (used in AFM experiments), suspensions were sonicated for 3 min (10 s on, 10 s off) at 50% power using a probe-type sonicator. For extrusion (used in calcein leakage and circular dichroism experiments), suspensions were extruded 19 times through 200 nm polycarbonate membranes (Millipore, Darmstadt, Germany) using a mini extruder (Avanti, Alabaster, AL, USA) after the freeze–thaw cycles.

### 4.4. Planar Lipid Bilayers

Microfluidic slides coated with Teflon film from Ionovation (Compact, Osnabrück, Germany) were employed for the formation of bilayer lipid membranes (BLMs). The lipid mixtures used for the bilayers were prepared in n-decane at a total concentration of 15 mg/mL. BLMs were created using the painting technique, with flexible plastic capillaries made from pipette tips, allowing for the careful maintenance of the aperture within the Teflon film without damage.

To replace the buffer in the chamber, 20 µL of a new solution was pipetted into the chamber simultaneously while aspirating 20 µL of the previous buffer. This process was repeated ten times, effectively replacing the buffer in the 100 µL compartments with excess new solution. The buffer composition for the BLM assembly consisted of 5 mM HEPES, 150 mM KCl, and 2 mM EDTA, which was adjusted to a pH of 7.40.

The criteria for initiating membrane work included the presence of a sharp meniscus–membrane border and a membrane capacitance of at least 40 pF. A HEKA EPC10USB amplifier was employed to measure membrane capacitance; we appli9ed a constant potential of 50 mV across the membrane and recorded the resulting current. Ag/AgCl electrodes (Ionovation GmbH, Bissendorf, Germany) with 2 M KCl/agarose bridges (1.5% *w*/*v* agarose) were used to apply the transmembrane voltage. Data were sampled at a frequency of 10 kHz, with a low-pass filter cutoff set at 5 kHz. The gain setting was configured to 0.2 mV/nA.

Measurements were conducted using a custom HEKA Patchmaster software (version 2x92) protocol, which recorded one-minute traces of conductance, interspersed with brief capacitance and current measurements. This procedure enabled periodic self-checks to rule out scenarios of membrane overflow (if capacitance became too low) or membrane disruption (if the circuit was shorted). Pore diameters were estimated by modeling the pores as cylindrical electrolytic conductors with a length equal to the bilayer thickness (4 nm) and the radius (r). The specific resistance (ρ) of the buffer solution was utilized to calculate resistance $R=\frac{\rho h}{\pi r^{2}}+\frac{\rho }{2r}$ by applying the observed pore current with a known applied potential.

### 4.5. Calcein Leakage Assay

Calcein was dissolved in phosphate-buffered saline (PBS; 10 mM phosphate buffer, 2.7 mM KCl, 137 mM NaCl, pH 7.40; PBS tablets from Sigma) at a self-quenching concentration of 40 mM. To obtain calcein-loaded liposomes, calcein-containing buffer was used for lipid film hydration, after which the previously mentioned freeze–thaw and extrusion procedures were conducted. Extruded liposomes were then separated from unencapsulated calcein using gel permeation chromatography on a Sephadex G-75 column with PBS as elution buffer. Fluorescence measurements were conducted using a Hitachi F-2500 spectrofluorometer (Tokyo, Japan) in time-scan mode, with an excitation wavelength of 495 nm and emission recorded at 510 nm. A baseline fluorescence (*I*_0_) was established by recording fluorescence for 1 min with the liposome sample, followed by the addition of the peptide, after which the fluorescence (*I_t_*) was recorded. To determine the maximum fluorescence (*I_max_*), 10 µL of 20% (*w*/*w*) Triton X-100 in water was added, and the sample fluorescence was recorded for an additional 1 min. After the addition of each component, the sample was thoroughly mixed in a cuvette by gently pipetting half of the sample volume back and forth at least 10 times. The percentage of calcein leakage was calculated using the equation $Leakage\%=\frac{I_{max}-I_{t}}{I_{max}-I_{0}}$.

### 4.6. Circular Dichroism

Circular dichroism (CD) measurements were performed using a Jasco J-1500 CD spectrometer (JASCO Corporation, Tokyo, Japan) with quartz cuvettes with a 2 mm path length. The spectra were recorded in the wavelength range of 190–250 nm. PBS was used as the buffer. For measurements involving peptides in the presence of liposomes, the peptide was added to liposomes suspended in PBS, and the sample was incubated for 60 min while being vortexed to ensure the complete redistribution of the peptide into the lipid bilayers prior to analysis.

### 4.7. Atomic Force Microscopy (AFM) Imaging and Force Spectroscopy

Lipid bilayers were prepared on freshly cleaved mica sheets using the liposome fusion method. To create the liquid chamber, a circular section was removed from the center of the bottom of a Petri dish, and a coverslip was glued to the underside. A small piece of mica (0.6 cm × 0.6 cm) was adhered to the center of the coverslip, and its surface layer was freshly cleaved using adhesive tape immediately before depositing the sonicated liposomes onto the mica.

AFM measurements were performed using an NTEGRA Prima atomic force microscope (NT-MDT, Moscow, Russia). For all experiments, silicon cantilevers (CSG-10) with a nominal spring constant of 0.12 N/m were used and calibrated prior to measurements using the thermal noise method available in the software. Sonicated liposomes (200 nm LUVs), at a concentration of 0.5 mg/mL, were applied to the cleaved mica surface and incubated for 30 min to form the lipid bilayer. After incubation, excess liposomes were removed by flushing the surface and gently washing off unbound vesicles. Before data acquisition, the system was equilibrated until the deflection signal stabilized.

Force spectroscopy measurements were performed to confirm bilayer formation using a fixed loading rate of 1 µm/s. For dynamic force spectroscopy experiments, the loading rate was varied between 0.1 and 3 µm/s. AFM imaging was carried out in tapping mode with a scanning rate of 1 Hz. All experiments were conducted in a buffer solution containing 150 mM KCl and 5 mM HEPES, at pH 7.4.

### 4.8. Data Analysis

AFM images were treated with Gwyddion 2.64 software. The force–distance curves were analyzed with Image Analysis 1.2.0.0 (Nova SPM 1.2). The data were plotted with OriginPro 2021.

## 5. Conclusions

In conclusion, the combination of AFM force spectroscopy, calcein leakage assays, and planar bilayer experiments provides a comprehensive picture of FP1-mediated membrane destabilization. Our findings not only demonstrate a reduction in the activation barrier for pore formation in DOPC membranes but also show that this mechanism extends to cholesterol-containing membranes, where FP1 facilitates intermediate pore formation and fusion pore catalysis. The similarities observed between FP1 and other amphipathic peptides suggest shared membrane-destabilizing mechanisms. Furthermore, by potentially regulating the threshold tension or elastic stress for membrane destabilization, cholesterol may play a critical role in ensuring successful viral entry at the appropriate stages of fusion. This underscores FP1’s critical versatility in viral fusion processes and presents opportunities for therapeutic intervention by targeting fusion peptide–membrane interactions.

## Figures and Tables

**Table 1:** Lipid compositions used in this work.

Composition	Molar Ratios	Abbreviation
DOPC	100	-
DOPC-Chol	70–30	-
DOPC-DOPS	90–10	-
DOPC:Chol:DOPE:DOPS	35.7:35:20.9:8.3 [41]	CMM

## Data Availability

The original contributions presented in this study are included in the article. Further inquiries can be directed to the corresponding author.
